# Reducing GHG emissions while improving diet quality: exploring the potential of reduced meat, cheese and alcoholic and soft drinks consumption at specific moments during the day

**DOI:** 10.1186/s12889-018-5132-3

**Published:** 2018-02-20

**Authors:** Mirjam E. van de Kamp, S. Marije Seves, Elisabeth H. M. Temme

**Affiliations:** The National Institute for Public Health and the Environment (RIVM), Postbus 1, 3720 BA Bilthoven, The Netherlands

**Keywords:** Sustainability, Scenario analysis, Greenhouse gas emissions, Diets

## Abstract

**Background:**

The typical Western diet is associated with high levels of greenhouse gas (GHG) emissions and with obesity and other diet-related diseases. This study aims to determine the impact of adjustments to the current diet at specific moments of food consumption, to lower GHG emissions and improve diet quality.

**Methods:**

Food consumption in the Netherlands was assessed by two non-consecutive 24-h recalls for adults aged 19–69 years (*n* = 2102). GHG emission of food consumption was evaluated with the use of life cycle assessments. The population was stratified by gender and according to tertiles of dietary GHG emission. Scenarios were developed to lower GHG emissions of people in the highest tertile of dietary GHG emission; 1) reducing red and processed meat consumed during dinner by 50% and 75%, 2) replacing 50% and 100% of alcoholic and soft drinks (including fruit and vegetable juice and mineral water) by tap water, 3) replacing cheese consumed in between meals by plant-based alternatives and 4) two combinations of these scenarios. Effects on GHG emission as well as nutrient content of the diet were assessed.

**Results:**

The mean habitual daily dietary GHG emission in the highest tertile of dietary GHG emission was 6.7 kg CO_2_-equivalents for men and 5.1 kg CO_2_-equivalents for women. The scenarios with reduced meat consumption and/or replacement of all alcoholic and soft drinks were most successful in reducing dietary GHG emissions (ranging from − 15% to − 34%) and also reduced saturated fatty acid intake and/or sugar intake. Both types of scenarios lead to reduced energy and iron intakes. Protein intake remained adequate.

**Conclusions:**

Reducing the consumption of red and processed meat during dinner and of soft and alcoholic drinks throughout the day leads to significantly lower dietary GHG emissions of people in the Netherlands in the highest tertile of dietary GHG emissions, while also having health benefits. For subgroups of the population not meeting energy or iron requirements as a result of these dietary changes, low GHG emission and nutritious replacement foods might be needed in order to meet energy and iron requirements.

## Background

The typical Western diet has a high environmental impact and is associated with obesity and other diet-related diseases, and therefore it can currently not be called sustainable. In the Netherlands, 50% of the adult population is overweight or obese [1]. The resulting chronic lifestyle diseases, such as cardiovascular disease and type 2 diabetes, cause major declines in quality of life and are primary causes of death [2, 3]. Like in many other countries, in the Netherlands the intake of fruits and vegetables is too low while the intake of saturated fatty acids (SFA) and sodium is too high [4, 5].

Food production and consumption systems are important drivers of greenhouse gas (GHG) emissions, with up to 30% of the worldwide emission of greenhouse gases being related to the production and consumption of food [6, 7]. Increasing the efficiency of food production systems is not enough to reduce GHG emissions to acceptable levels: our consumption patterns will have to change as well [6, 8]. Shifting to healthier diets will only result in reduced GHG emissions if consumers choose healthy foods with a relatively low impact on GHG emissions [9, 10]. At a comparable level of dietary energy intake, GHG emissions of diets may vary substantially. For example, diets containing 2000 kcal per day may vary from ~ 2 to > 12 kg CO_2_-equivalents (CO_2_-eq) per day [11].

Animal-based foods are generally the most important contributors to resource use and dietary GHG emissions [12]. This is also true for Dutch food consumption patterns [13], where almost half of the GHG emissions of food consumption can be attributed to meat and dairy consumption. The total consumption of all beverages also contributes considerably to dietary GHG emissions [13, 14]. Previous research in the Netherlands has already quantified the potential effects of replacing meat and dairy with plant-based foods on GHG emissions and nutrient content of the diet, for the general adult population [15]. Especially the diets of those with high dietary GHG emissions hold a large potential for improvement. To our knowledge, little research has been done to estimate the effects of dietary changes to lower GHG emissions in this specific group, on GHG emissions as well as intake of selected nutrients. Since people with relatively high dietary GHG emissions also have significantly higher energy intakes [13], reducing the intake of high GHG emission foods such as meat might be preferable over replacing these foods. If there is overconsumption in this subgroup of the population, reducing food (and therefore energy) intake could be beneficial for health as well as the reduction of GHG emissions.

This scenario study aims to provide suggestions to reduce the GHG emission of diets with high associated GHG emissions, while simultaneously improving the nutrient content of these diets. The reference scenario is the diet of people in the highest tertile of dietary GHG emissions in the Netherlands. For specific moments of consumption during the day, the intake of foods with the highest associated GHG emissions is either reduced or replaced with healthier alternatives with lower GHG emissions, by means of several scenarios. The results of this study can provide valuable knowledge for the design of policies and interventions directed at stimulating healthy dietary patterns with reduced GHG emissions, for people with relatively high dietary GHG emissions.

## Methods

### Food consumption data

The consumption data were obtained from the most recent food consumption survey in the Netherlands, the Dutch National Food Consumption Survey (DNFCS) 2007–2010, described in more detail elsewhere [4]. In short, food consumption was based on two 24-h recalls that were conducted by dietitians on independent days about four weeks apart with use of the EPIC-Soft® program (currently known as GloboDiet). During the dietary recalls, the moment of food consumption was registered. All consumptions were labelled as one of seven food consumption occasions, distinguishing between the three main meals (breakfast, lunch, dinner) and all moments in between meals (before breakfast, in between breakfast and lunch, in between lunch and dinner, after dinner). Food composition data were taken from the extended Dutch Food Composition Table (NEVO-Table 2011/3.0) [16]. The study population is a representative sample of the Dutch population with respect to age, gender, region, degree of urbanisation and educational level. The overall response was 69% (*n* = 3819) [4]. Included in this study were men and women aged 19–69 years. Excluded were those who consumed only meal replacements (for weight reduction) (*n* = 1) or those who were lactating and thus had different nutrient requirements (*n* = 4). Calculations were based on 2102 individuals (1055 men and 1047 women).

### General questionnaire

A general questionnaire was used to cover various socio-demographic and lifestyle factors such as physical activity and educational level as described by Van Rossum et al. [4]. Physical activity data were obtained according to the SQUASH (Short QUestionnaire to ASsess Health enhancing physical activity) questionnaire [17]. Based on the information in the questionnaire, time spent on physical activity was taken together (according to the SQUASH manual) and calculated as MET x h/week [4]. MET are metabolic equivalents to assess physical activity levels [18]. Information on educational level was aggregated into low (primary education/lower vocational education/low or intermediate secondary education), middle (intermediate vocational education/higher secondary education) and high (higher vocational education/university). Net household income was aggregated into three categories: < 1700 euro/month, 1700–2900 euro/month and > 2900 euro/month. Ethnicity was based on self-reported information on the participants’ native country. Furthermore, height and weight were self-reported during the 24-h recall interviews and average values for both height and weight were calculated based on the two interviews. Body mass index (BMI) was determined as the average body weight (in kg) divided by average height squared (kg/m^2^). Subsequently, these BMIs were classified according to the WHO cut-off points for adults [19]. Estimates of Basal Metabolic Rate (BMR) were calculated from standard equations based on weight, age and sex [20].

### Greenhouse gas emissions

The emission of greenhouse gases (in kg CO_2_-eq per day) is an indicator for global warming potential [21]. In order to estimate the GHG emissions associated with different foods, life cycle assessments (LCAs) were performed by Blonk Consultants (dataset version 2012) using Agri-footprint [22]. These LCAs include the entire life cycle of a product (i.e. cradle to plate assessment), from primary production, processing, use of packaging and transport to storage (including energy for cooling and freezing of products) and preparation of food. Land use change was not included in the LCAs. In addition, transport by consumers (i.e. from grocery store to the home) was not included due to large variation. Since this transport will vary between persons but will mostly be the same for different food products, this exclusion will not influence the comparisons in this study. The GHG emissions associated with the preparation of foods in the consumer phase was based on the average cooking time for each product and an energy mix representative for the Dutch market (based on household energy use for cooking). Food waste was included by using food group specific percentages for avoidable and unavoidable food losses throughout the food chain, including the consumer phase. For production processes that lead to more than one food product, economic allocation was used. Life-cycle inventory data were collected from primary sources and literature and were representative for the Dutch situation. The time horizon for the GHG emission calculations was 100 years. GHG emission data were accessible for 306 frequently consumed food items covering > 80% of the total food weight (in grams per day) consumed in the DNFCS 2007–2010. An experienced dietitian extrapolated the available LCA data to > 1300 other reported food items, based on similarities in type of food, production system and ingredient composition. The mean GHG emissions for each food group have previously been described in more detail elsewhere [13]. The LCA data were combined with the food consumption data to calculate the GHG emissions of the diet.

### Scenarios

The log transformed mean daily dietary GHG emission from the two recalled days was used to classify the population according to low (≤ P33), intermediate (> P33 and ≤ P66) and high (> P66) dietary GHG emissions. Several scenarios were developed aimed at reducing food-related GHG emissions and improving diet quality for men and women in the highest tertile of dietary GHG emission (=reference). The diet of people in the highest tertile of dietary GHG emissions has previously been described in more detail elsewhere [13]. In the scenarios, the consumption of animal products (meat and cheese) and drinks (alcoholic drinks and soft drinks including mineral water) was reduced or replaced by other foods during those moments of consumption in which these foods had the highest impact (Table 1). Replacement foods were well-accepted foods in the Netherlands and were of similar use and equal quantity (in grams) as the original food consumed in the DNFCS 2007–2010. Moreover, replacement foods had to have lower GHG emissions per kg product and had to simultaneously contribute to a favourable shift in nutrient intakes. Foods other than defined in the scenarios were not replaced and consumption of these foods was assumed not to change. To model the replacement scenarios, SAS software (version 9.4, SAS Institute Inc., Cary, NC, USA) was used to assign a random number between 0 and 1 to all consumptions of products eligible for replacement as consumed in the reference scenario, for each person, each observed day and each consumption on those days.

**Table 1:** Scenarios to reduce dietary GHG emission for people in highest tertile of dietary GHG emission

Foods	Consumption occasion	Reductions/replacements	Scenario
Cheese	In between meals	Cheese without bread^a^ 50% replaced by nuts (mixed, unsalted) 50% replaced by cherry tomatoes	‘Cheese’
		Cheese with bread^a^ 50% replaced by peanut butter 50% replaced by vegetable sandwich spread	
Meat	Dinner	Red/processed meat consumption reduced by 50% (not replaced)	‘Meat50’
		Red/processed meat consumption reduced by 75% (not replaced)	‘Meat75’
Drinks	All day	Alcoholic drinks and soft drinks^b^ 50% replaced by tap water	‘Water50’
		Alcoholic drinks and soft drinks^b^ 100% replaced by tap water	‘Water100’
Combinations		Combination of Cheese + Meat50 + Water50	‘Combi50’
		Combination of Cheese + Meat75 + Water100	‘Combimax’

^a^Random replacement of consumptions on a population level by plant-based substitutes of the same quantity (in grams)

^b^Soft drinks were defined as all cold non-alcoholic drinks, excluding tap water and dairy drinks but including fruit drinks, fruit nectars, vegetable juices, carbonated drinks, syrups, lemonades, vitamin waters, mineral waters, energy drinks, sports drinks and iced tea

First, in the scenario ‘cheese’, all cheese consumed in between meals was randomly replaced by plant-based alternatives. Cheese consumption was identified as consumed with or without bread (including rusk bread, crispbread and products such as rolls, crackers and toast). When cheese was consumed in combination with bread (or comparable products), it was replaced by one of two other sandwich spreads, i.e. by peanut butter if the randomly assigned number fell in the range 0.0–0.5 or by vegetable sandwich spread if the number fell in the range > 0.5–1.0. Cheese consumed without bread was replaced by either cherry tomatoes or mixed nuts (unsalted) using the same methodology.

In the second and third scenario the amount of red and processed meat consumed during dinner, was reduced by 50% (‘meat50’) or 75% (‘meat75’). Red meat was defined as unprocessed beef, pork, veal, mutton/lamb, horse, goat and game and excluding poultry, fish or egg. Processed meat was defined as any meat preserved by smoking, curing, salting or by adding chemical preservatives, such as bacon and sausages, excluding fish or egg. Processed poultry products were included in the ‘processed meat’ category. When meat products were reduced by 50% or 75%, all consumed quantities in the reference scenario, corresponding nutrient intakes and GHG emission, were multiplied by 0.5 or 0.25 respectively.

The fourth and fifth scenario include the replacement of 50% (‘water50’) or 100% (‘water100’) of soft drinks (including mineral water and fruit or vegetable juices) and alcoholic drinks by tap water, for all moments of consumption. Soft drinks were defined as all cold non-alcoholic drinks, excluding tap water and dairy drinks but including fruit drinks, fruit nectars, vegetable juices, carbonated drinks, syrups, lemonades, vitamin waters, mineral waters, energy drinks, sports drinks and iced teas. Soft drinks include both the regular as well as the light/diet versions of these drinks, since these were assumed to have equal GHG emissions. Coffee (including coffee alternatives), tea (including herbal tea), dairy drinks and tap water were not replaced. In the scenario ‘water50’, the consumed soft drinks and alcoholic drinks were replaced by tap water if the allocated random number fell in the range 0.0–0.5, and were not replaced if the number fell in the range > 0.5–1.0. In the scenario ‘water100’, all soft drinks and alcoholic drinks were replaced by tap water.

Finally, two combined scenarios were developed: an intermediate (‘combi50’) and a maximum (‘combimax’) combination scenario. The intermediate scenario included all reductions and replacements as defined in the scenarios ‘cheese’, ‘meat50’ and ‘water50’. The maximum scenario included all reductions and replacements as defined in the scenarios ‘cheese’, ‘meat75’ and ‘water100’.

### Statistical analyses

Descriptive statistics (medians, interquartile ranges and percentages of the population) were calculated for several baseline characteristics of participants in the low, intermediate and high tertile of dietary GHG emission. Differences between groups were evaluated using chi-square tests for categorical variables and Kruskal-Wallis tests for continuous variables (since the assumptions for ANOVA were not met). Bonferroni corrections were applied for multiple comparisons. For these calculations, SAS software (version 9.4, SAS Institute Inc., Cary, NC, USA) was used. Dietary GHG emission and daily intake of energy, protein, mono- and disaccharides (as proxy for sugar), SFA, sodium and iron were calculated for each scenario (including the reference) for men and women separately, in order to investigate the effects of our replacement scenarios on nutrient intakes. Energy intake was also calculated for the low and intermediate tertile of dietary GHG emission. Since the main interest is in the long-term nutrient intake, the so-called habitual intake, statistical modelling was applied to the two 24-h recalls per person to account for the intra-individual variation (day-to-day variation). Habitual intake distributions were estimated with SPADE (Statistical Program to Assess Dietary Exposure, version 3.1) using the 1-part model for components consumed on a daily basis for all subjects [23]. A weighing factor was included to account for small deviances in socio-demographic characteristics, days of the week and season of data collection, to make the results representative for the Dutch adult population, for every day of the week and every season. 95% confidence intervals (CI) around the mean point estimate were computed using the bootstrap method with 1000 iterations. Significant differences between reference and reduction/replacement scenarios were evaluated by non-overlapping 95% CI.

To evaluate adequacy of protein intake, the population’s habitual protein intake distribution was compared to the Estimated Average Requirement (EAR) as established by the Health Council of the Netherlands, based on reference body weights for the Dutch population [24]. For this purpose, the EAR cut-point method was used [25]. Due to methodological constraints it was not possible to evaluate energy and iron intake via the EAR cut-point method.

## Results

In the highest tertile of dietary GHG emission for women, age was slightly higher compared to the intermediate and low tertile of dietary GHG emission (Table 2). There were more non-Dutch participants in the lowest tertile of dietary GHG emission for women. The proportion of participants with overweight (including obesity) was not significantly different between the tertiles of dietary GHG emission, for men as well as women. There were no significant differences in BMR between groups. Men in the lowest tertile of dietary GHG emission were less physically active (based on the MET score) than participants in the other tertiles, but the MET scores of men in the intermediate and high tertile were not significantly different. There were no significant differences in MET scores for women in the three tertiles of dietary GHG emission.

**Table 2:** Participant characteristics by tertile of dietary GHG emission (median (IQR))

	Men			Women		
	Low (*n* = 352)	Intermediate (*n* = 352)	High (*n* = 351)	Low (*n* = 348)	Intermediate (*n* = 350)	High (*n* = 349)
Tertile cut-off points						
GHG emission (kg CO_2_-eq/d)^a^	≤3.9	3.9-5.1	≥ 5.1	≤3.0	3.0–4.0	≥ 4.0
Characteristics						
Age (years)	40 (28–56)	39 (28–55)	42 (29–56)	38 (27–54)	39 (28–54)	44 (30–58)
Low educational level (%)^b^	29	27	36	40	35	36
Net household income						
< 1700 euro/month (%)	29	27	28	38	37	32
1700–2900 euro/month (%)	51	50	48	42	45	50
> 2900 euro/month (%)	20	23	25	20	18	19
Dutch ethnicity (%)	96	97	98	93	96	97
BMI^c^						
Overweight (%)	41	35	44	25	30	33
Obesity (%)	15	16	10	22	22	21
MET score (hours/week)	142 (88–197)	160 (111–223)	167 (112–229)	146 (94–210)	154 (101–211)	157 (117–226)
BMR (kJ/h/kg body mass)^d^	7.7 (7.2–8.3)	7.7 (7.3–8.3)	7.7 (7.3–8.1)	6.0 (5.6–6.5)	6.1 (5.8–6.6)	6.0 (5.7–6.6)

*BMR* basal metabolic rate, *CO*_*2*_*-eq* carbon dioxide equivalent, *GHG* greenhouse gas, *MET* metabolic equivalent

^a^Average GHG emission for a day’s consumption based on two 24-h recalls used to define low (≤ P33), intermediate (> P33 and ≤ P66) and high (> P66) dietary GHG emission

^b^Low education was defined as primary education/lower vocational education/low or intermediate secondary education

^c^Overweight was defined as a BMI ≥25 and < 30; and obesity as a BMI ≥30 [19]

^d^BMR calculated from standard equations based on weight, age and sex [20]

### GHG emission of daily food consumption

In the highest tertile of dietary GHG emission, food consumption is associated with a mean emission of 6.7 kg CO_2_-eq per day for men and 5.1 kg CO_2_-eq per day for women. People with higher dietary GHG emissions have higher energy intakes. Habitual energy intake in the highest tertile of dietary GHG emissions (mean intake of men = 3112 kcal/day and mean intake of women = 2287 kcal/day) is significantly higher than in the intermediate tertile (mean intake of men = 2658 kcal/day and mean intake of women = 1957 kcal/day). Similarly, energy intake in the intermediate tertile of dietary GHG emissions is significantly higher than in the lowest tertile (mean intake of men = 2090 kcal/day and mean intake of women = 1641 kcal/day). The higher dietary GHG emission is mainly associated with a higher consumption of meat. Meat consumption contributes 42% and 39% to daily dietary GHG emission in the highest tertile of dietary GHG emission for men and women respectively, compared to 31% and 28% in the intermediate tertile.

### GHG emission per consumption occasion and food group

**Fig. 1:**
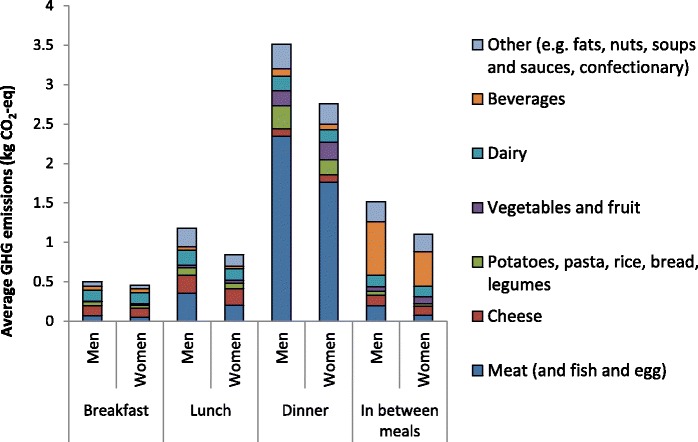
Mean daily GHG emission (kg CO_2_-eq) of food groups per consumption occasion in reference scenario

In the reference scenario, dietary GHG emission was highest during dinner (mainly caused by meat consumption) and for consumptions in between meals (mainly caused by the consumption of beverages; Fig. 1). The foods contributing most to dietary GHG emissions were meat products (around 40%), dairy products including milk and cheese (around 20%, of which 10% for cheese and 10% for other dairy products), and beverages (around 10%). The mean consumption of meat (excluding fish and egg) was 185 g per day for men and 119 g per day for women, of which the major part was consumed during dinner (70% for men and 78% for women) and consisted of red and/or processed meat (almost 90%).

Cheese consumed throughout the day contributed 9% and 10% to dietary GHG emission of men and women respectively. Daily cheese consumption was 48 g for men and 43 g for women. Most of the cheese was consumed during lunch (~ 40%) and during breakfast and in between meals (both ~ 20%). About half of the cheese consumption in between meals was consumed in combination with bread or comparable products. Beverages (excluding milk) were mainly consumed in between meals, and especially during the evening. More than half of the GHG emission caused by the consumption of beverages (excluding milk) was from consumptions during the evening. The total consumption of beverages (excluding milk) contributed 13% to the dietary GHG emission of men and 12% to the dietary GHG emission of women. Daily consumption of all drinks excluding dairy, tap and mineral water, and coffee and tea, was about 1 l for men and about half a litre for women. Men consumed almost four times more alcoholic drinks than did women.

### Scenarios

The ‘water100’, ‘meat50’, ‘meat75’, ‘combi50’ and ‘combimax’ scenarios all significantly reduced dietary GHG emission for men and women in the highest tertile of dietary GHG emission (Table 3). The scenarios including a reduction of meat consumed during dinner hold the largest reduction potential in terms of dietary GHG emission; ranging from approximately − 15% in the ‘meat50’ scenario to up to − 34% in the ‘combimax’ scenario.

**Table 3:** Mean daily habitual dietary GHG emission and nutrient intakes (with 95% CI), for all scenarios

		GHG emission (kg CO_2_-eq)	Energy (Kcal)	Protein (g)	Mono- and disaccharides (g)	SFA (g)	Sodium (g)^a^	Iron (mg)
Reference	Men	6.7 (6.5–6.8)	3112 (3041–3185)	120 (117–123)	139 (133–145)	46 (44–48)	3.7 (3.6–3.8)	14.2 (13.8–14.6)
	Women	5.1 (5.0–5.3)	2287 (2233–2345)	92 (89–94)	117 (112–122)	36 (35–37)	2.8 (2.7–2.9)	11.6 (11.3–12.0)
Cheese	Men	6.6 (6.4–6.7)	3116 (3044–3188)	119 (116–121)	140 (134–146)	44 (43–46)	3.7 (3.6–3.8)	14.3 (14.0–14.7)
	Women	5.0 (4.9–5.2)	2292 (2237–2351)	91 (88–93)	118 (113–122)	34 (33–36)	2.8 (2.7–2.9)	11.7 (11.4–12.1)
Water50	Men	6.4 (6.2–6.5)	2910 (2848–2979)*	119 (116–121)	122 (117–128)*	46 (44–48)	3.7 (3.6–3.8)	13.8 (13.4–14.2)
	Women	5.0 (4.8–5.1)	2186 (2135–2242)	91 (89–93)	103 (99–107)*	36 (34–37)	2.8 (2.7–2.9)	11.2 (10.9–11.5)
Water100	Men	6.1 (5.9–6.2)*	2694 (2629–2758)*	118 (115–120)	104 (99–109)*	46 (44–48)	3.7 (3.6–3.8)	13.4 (13.1–13.8)
	Women	4.8 (4.6–4.9)*	2073 (2024–2127)*	91 (88–93)	88 (84–92)*	36 (34–37)	2.8 (2.7–2.9)	10.7 (10.4–11.1)*
Meat50	Men	5.6 (5.5–5.7)*	2976 (2908–3048)	106 (103–108)*	139 (133–145)	43 (41–44)	3.5 (3.4–3.7)	13.0 (12.7–13.4)*
	Women	4.4 (4.3–4.5)*	2191(2140-2250)	81 (79–83)*	117 (112–122)	33 (32–35)	2.7 (2.6–2.8)	10.8 (10.5–11.2)*
Meat75	Men	5.1 (5.0–5.2)*	2909 (2839–2978)*	99 (96–101)*	139 (133–145)	41 (40–43)*	3.5 (3.3–3.6)	12.5 (12.1–12.9)*
	Women	4.0 (3.9–4.1)*	2143 (2092–2202)*	76 (74–78)*	117 (112–122)	32 (31–34)*	2.6 (2.5–2.7)	10.4 (10.1–10.8)*
Combi50^b^	Men	5.3 (5.1–5.4)*	2809 (2745–2878)*	104 (101–106)*	125 (120–130)*	41 (40–43)*	3.5 (3.4–3.6)	12.7 (12.4–13.1)*
	Women	4.1 (4.1–4.2)*	2114 (2065–2171)*	80 (78–82)*	106 (102–111)*	32 (31–33)*	2.6 (2.5–2.7)	10.5 (10.2–10.8)*
Combimax^c^	Men	4.4 (4.3–4.5)*	2494 (2430–2556)*	95 (93–97)*	105 (100–110)*	39 (38–41)*	3.4 (3.2–3.5)*	11.7 (11.4–12.1)*
	Women	3.5 (3.4–3.6)*	1934 (1885–1988)*	75 (72–77)*	89 (85–92)*	31 (30–32)*	2.5 (2.5–2.6)*	9.6 (9.3–9.9)*

*GHG* greenhouse gas, *CI* confidence interval, *SFA* saturated fatty acids

The 95% confidence intervals are based on 1000 bootstrap samples

^a^Excluding added sodium during cooking or at the table

^b^Combi50 combines scenario cheese, water50 and meat50

^c^Combimax combines scenario cheese, water100 and meat75

*Significantly different from the reference based on the 95%-CI around the mean point estimate

The replacement of cheese consumed in between meals by plant-based substitutes, as well as the replacement of 50% or 100% of soft drinks and alcoholic drinks consumed by tap water, both led to a reduction in dietary GHG emission of < 10% compared to the reference.

Replacing cheese in between meals by plant-based substitutes did not significantly alter the intake of the selected nutrients (Table 3). Full replacement (100%) of soft drinks and alcoholic drinks by tap water significantly decreased energy and mono- and disaccharides intakes (as proxy for sugar) of men and women, and iron intake of women only. When these drinks were partly (50%) replaced, there was no significant difference in iron intakes, the reduction in energy intake was only significant for men, and the decrease in mono- and disaccharides intakes remained significant for both men and women. A reduction of red and processed meat consumed during dinner with 75%, resulted in significant decreases in energy, SFA, protein and iron intake for men as well as women. Both combination scenarios led to a significant decrease in energy, protein, mono- and disaccharides, SFA and iron intakes compared to the reference. The ‘combimax’ scenario additionally resulted in a significant 8–11% reduction in sodium intake. Protein intake remained adequate in all scenarios; the proportion of adults with habitual intakes below the corresponding EAR remained 0% for both men and women.

In the ‘combimax’ scenario, the remaining average daily meat consumption was 100 g for men and 60 g for women. Average cheese consumption was 37 g per day for men and 34 g per day for women when all cheese consumption in between meals was replaced by plant-based alternatives. In this scenario, consumption of all soft drinks and alcoholic drinks was replaced by tap water, so consumption of those drinks was 0 g per day.

## Discussion

This study aimed to provide suggestions for dietary change to reduce GHG emissions and at the same time improve nutrient quality of diets associated with high GHG emissions among Dutch adults. Several dietary changes at specific moments of food consumption can reduce GHG emission of these diets by up to one third, while lowering energy, SFA and sugar intakes, but also iron intakes. Considering that 54% of men and women in our study population were overweight, reduced energy intake will be beneficial for most, but for part of the population the scenarios could lead to energy and iron intakes below requirements. In those cases, increased consumption of low GHG emission and nutritious replacement foods will be needed.

In the Dutch diet, the GHG emission varies per consumption occasion. In the highest tertile of dietary GHG emission, dinner is associated with the highest GHG emission, and consumptions in between meals are associated with the second-to-highest emissions. Meat consumption contributes most to the GHG emission of dinner, while beverages contribute most to the GHG emission of consumptions in between meals. Dairy (including cheese) is an important contributor to total daily dietary GHG emission throughout the day.

The most effective single dietary change to reduce GHG emissions in this study was to reduce the consumption of meat, with a 75% reduction in red and/or processed meat during dinner resulting in a 24% reduction in GHG emissions for men and a 22% reduction for women. Average daily meat consumption in the highest tertile of dietary GHG emission of our study population was 185 g for men and 119 g for women, of which almost 90% was red and/or processed meat. This is well above the recommended maximum intake of 500 g meat per week (or 71 g per day), of which a maximum of 300 g (or 43 g per day) may be red meat (60%), according to the current Dutch food based dietary guidelines [26]. Reducing the consumption of meat is therefore in line with the food based dietary guidelines. In the ‘meat75’ and ‘combimax’ scenarios, remaining weekly meat consumption is 700 g (of which 567 g red and/or processed meat) for men and 420 g (of which 308 g red and/or processed meat) for women. Meat consumption of women is slightly lower than the recommended maximum in the food based dietary guidelines, but protein intake remained adequate. The ‘meat75’ and ‘combimax’ scenarios resulted in decreased saturated fatty acid intakes. Reducing the consumption of red and/or processed meat may also lower the risk for chronic diseases, as research shows that every 50 g portion of processed meat eaten daily increases the risk of colorectal cancer by about 18% [27]. In the scenario that reduced meat consumption during dinner with 75%, the average consumption of red and/or processed meat was reduced with 85 g/day for men and 59 g/day for women.

Replacing the consumption of all alcoholic drinks and soft drinks (including mineral water and fruit and vegetable juices) with tap water was also successful in reducing dietary GHG emission. At the same time, the intake of energy and mono- and disaccharides (as proxy for sugar) was reduced significantly and for women there was also a significant and relevant reduction in iron intakes. This can be explained by the fact that women drink relatively more iron-containing drinks. For example, 61% of all alcoholic drinks consumed by women is wine, while for men 87% of alcoholic drinks consumed is beer. According to the food composition table used for this study, beer contains no iron while wine contains 0.6 mg per 100 g. Women also drink relatively more fruit juices (containing some iron) while men drink more other soft drinks. However, because the consumption of both alcoholic drinks and fruit juices is discouraged in dietary guidelines, these foods are not the most appropriate dietary iron sources. Dietary iron could be obtained from types of foods with a more healthy nutritional profile, containing other essential nutrients while the content of nutrients that are considered unhealthy is low. When drinks were partly (50%) replaced, iron intakes were not compromised and there was a significant decrease in mono- and disaccharides intakes for men and women, while energy intake was reduced for men only. This can be explained by the fact that men have a higher consumption of these drinks in the reference scenario. The ‘water50’ scenario did however not lead to significant GHG emission reductions. The consumption of beverages is highest in between meals. Consuming water instead of sugar-containing or acidic beverages in between meals has the additional benefit of a less frequent exposure to foods that can damage tooth enamel (when tap water is consumed without food) [28].

Our scenarios show that reducing the consumption of soft and alcoholic drinks and of red and processed meat will lead to lower dietary GHG emissions, even if meat intake is only reduced at dinner. These dietary changes are also beneficial for health because they bring food consumption more in line with food based dietary guidelines [26] and will reduce SFA and sugar intakes. However, these changes will also lead to lower energy and iron intakes. Considering that 54% of men and women in the highest tertile of dietary GHG emissions were overweight or obese (see Table 2), reduced energy intake is beneficial for the majority of the population under study. Estimated average energy requirements as previously calculated [29] for the Dutch food based dietary guidelines [26] range from 2430 kcal/day to 2790 kcal/day for men aged 19–69 years, and from 1790 kcal/day to 2020 kcal/day for women aged 19–69 years. The mean energy intake in most of our scenarios is still higher than these average requirements. However, for part of the population the scenarios could lead to energy intakes below requirements. We were unable to assess energy adequacy and thus the proportion of the population with an intake below the requirement, since we could not determine individual energy requirements sufficiently accurate from the DNFCS 2007–2010 data available. Individual energy requirements may vary widely depending on BMR and level of physical activity. Iron adequacy could also not be assessed: the EAR cut-point method cannot be used because the requirement distribution for iron is not symmetric [25]. Because the iron requirement distribution is unknown for our study population, it was also not possible to use the probability method to assess adequacy [25]. The Nordic Council [30] has established an EAR for iron of 7 mg/day for men aged 19–69, 10 mg/day for women aged 19–50 and 6 mg for women aged 51–69 (postmenopausal women). Mean iron intake of men in our scenarios is well above the EAR, and for women mean intakes are close to the EAR for menstruating women. Since mostly women of childbearing age are at risk of having inadequate iron intakes [4], iron intake reductions as observed in our scenarios are undesirable for this subgroup. However, because iron inadequacy already occurs in this subgroup [4], the scenarios in this study might increase the proportion of women with inadequate iron intakes but do not introduce a new problem. Effort is needed to ensure adequate iron intakes of women of childbearing age, regardless of whether meat and alcoholic and soft drink consumption are maintained or reduced. For people with energy and/or iron intakes below requirements as a result of the dietary changes in our scenarios, the reduced meat and soft and alcoholic drinks intake should be compensated for by increased intake of other low GHG emission, healthy foods. Previous research in the general Dutch population has shown that replacing meat and dairy with plant-based alternatives can significantly reduce GHG emissions while increasing estimated iron intakes [15]. However, the iron from plant-based sources is of lower bioavailability and future research should take this into account in order to determine if iron intake from plant-based foods replacing meat is adequate to meet requirements [31].

Combining the meat reduction and drinks replacement scenarios into the ‘combimax’ scenario leads to the highest reductions in dietary GHG emission. The reduction in mean habitual daily dietary GHG emission is 34% for men and 31% for women in the ‘combimax’ scenario. These combination scenarios also include the replacement of all cheese consumed in between meals by plant-based alternatives. This individual change led to a small but non-significant decrease in dietary GHG emission. The GHG emission reductions in this scenario are therefore mainly attributable to the reduction in meat consumption and the replacement of alcoholic drinks and soft drinks. These results are in line with previous research. Green et al. have shown that for a UK population, the consumption of meat (especially red meat) and soft drinks should decrease in order to achieve GHG emission reductions [14]. Similarly, Horgan et al. showed that for sustainable diets, the consumption of soft drinks and red and/or processed meat should be reduced in the diets of most individuals in their UK study population [9]. Previous research for the Dutch situation has shown that vegetarian and vegan diets are most effective in reducing GHG emissions [32, 33].

What makes our approach novel is that we have taken consumption occasion into account. Of all meat consumed, the vast majority is consumed during dinner. Important reductions in dietary GHG emission as a result from reduced meat consumption can be achieved by focussing only on that consumption occasion. Previous research in the Dutch population has shown that willingness to change the diet is generally low, and that people are most willing to adopt changes that do not cost money and that are relatively effortless [34]. Both reducing meat consumption during dinner and replacing alcoholic drinks and soft drinks by tap water in between meals will save money. Both changes are also less drastic than eliminating meat from the diet entirely or replacing all drinks (including coffee and tea) by tap water, which might make it relatively easy to implement these changes. Indeed, Sijtsema et al. have showed that willingness to limit meat consumption is higher than willingness to change to a vegetarian diet [34] The first results from the Dutch National Food Consumption Survey 2012–2016 show that there has been a decrease in meat consumption in the Netherlands [35], so possibly further reductions in meat consumption would be acceptable to the population. Consumption of alcoholic drinks has decreased as well, while intake of non-alcoholic drinks increased. However, the latter was caused by increased consumption of tea, coffee and water [35]. These changes are in the direction also proposed in the scenarios in this study. However, it is unknown if the reductions in meat and soft and alcoholic drinks consumption in the scenarios are indeed acceptable to the subgroup of the population included in this study. Taste and food preferences were not assessed. Also, if reduced meat intake during dinner would be compensated for by increased intakes of meat during other moments of food consumption, this will affect the GHG emission reductions. Similarly, if a reduced consumption of meat during dinner would be compensated for by increased consumption of other foods during dinner, it depends on the GHG emissions associated with the replacement foods what the final effect on dietary GHG emissions will be.

The strengths of this study include the focus on GHG emissions as well as nutrient content of the diet, the fact that the scenarios are based on current food consumption using data from the most recent DNFCS (based on a large and representative group of Dutch adults), and the fact that the suggested dietary changes are based on the highest potential GHG emission reductions at specific moments of food consumption.

A limitation of our study could be that we relied on GHG emission data only. While this indicator is highly correlated to some other indicators of environmental impact [36], it does not mean that a reduction in GHG emissions leads to similar reductions in all other indicators of environmental impact. Additional research would be needed to quantify the effect of the scenarios included in this study on other indicators of environmental impact, such as land or water use. Future research would also be necessary to determine how the subgroup of the population included in this study could be moved to adopt the dietary changes proposed, as it is very difficult to achieve dietary change. A long-term approach is needed to change consumer values, and a positive attitude towards sustainable food consumption may not be enough to change dietary behaviour [37]. Health-related arguments may be more effective than environmental motives to promote sustainable consumption [38], but coupling these arguments in communication strategies or in dietary guidelines may be worthwhile since both arguments may appeal to different segments of the population. As this study shows some dietary changes will be beneficial for both health and the environment, at least for people in the highest tertile of dietary GHG emissions. Therefore, an effort should be made to further integrate environmental sustainability into dietary guidelines. Even more than individual approaches, there is a need for social and institutional changes that facilitate environmentally friendly food consumption [37].

The scenarios in this study were focussed on people in the highest tertile of dietary GHG emission, since that subgroup of the population holds the largest potential for dietary GHG emission reductions. Previous research has shown that for the entire Dutch population, meat and cheese combined are the largest contributor to dietary GHG emission, followed by drinks [13]. Out of all types of drinks, soft drinks are an important contributor to GHG emission for the whole population, and alcoholic drinks are an important contributor to GHG emission of adult men specifically. It is, therefore, expected that the dietary changes proposed in this study will also lead to reductions in the GHG emission of people in the lowest or intermediate tertile of dietary GHG emission. However, the effect of the scenarios included in this study on nutrient intakes of the general population are unknown, and therefore the results of this study cannot be extrapolated to other groups. Additional research will be necessary to quantify how the scenarios in this study affect nutrient intakes of people with lower dietary GHG emissions.

## Conclusion

Reducing the consumption of red and processed meat during dinner and of soft and alcoholic drinks throughout the day leads to significantly lower dietary GHG emissions of people in the Netherlands in the highest tertile of dietary GHG emissions, while also having health benefits. For subgroups of the population not meeting energy or iron requirements as a result of these dietary changes, low GHG emission and nutritious replacement foods might be needed in order to meet energy and iron requirements.

## Abbreviations

BMI: Body mass index
BMR: Basal metabolic rate
CI: Confidence interval
CO_2_-eq: CO_2_-equivalent
DNFCS: Dutch National Food Consumption Survey
EAR: Estimated average requirement
GHG: Greenhouse gas
LCA: Life cycle assessment
MET: Metabolic equivalents
SFA: Saturated fatty acids
